# Identification and validation of key miRNAs and miRNA–mRNA regulatory network associated with uterine involution in postpartum Kazakh sheep

**DOI:** 10.5194/aab-64-119-2021

**Published:** 2021-04-23

**Authors:** Heng Yang, Lin Fu, Qifeng Luo, Licai Li, Fangling Zheng, Jiayu Wen, Chenjing Li, Xingxiu Luo, Zongsheng Zhao, Huihao Xu

**Affiliations:** 1 College of Veterinary Medicine, Southwest University, Rongchang 402460, Chongqing, China; 2 Immunology Research Center, Medical Research Institute, Southwest University, Rongchang 402460, Chongqing, China; 3 Chongqing Academy of Animal Sciences, Rongchang 402460, Chongqing, China; 4 College of Animal Science and Technology, Shihezi University, Shihezi 832000, Xinjiang, China

## Abstract

MicroRNAs (miRNAs) are widely expressed in different mammalian tissues and exert their biological effects through corresponding target genes. miRNA target genes can be rapidly and efficiently identified and screened by combining bioinformatics prediction and experimental validation. To investigate the possible molecular regulatory mechanisms involving miRNAs during uterine involution in postpartum ewes, we used Illumina HiSeq sequencing technology to screen for the number and characteristics of miRNAs in faster uterine involution and normal uterine involution group. A total of 118 differentially expressed miRNAs, including 33 known miRNAs and 85 new miRNAs, were identified in the hypothalamic library, whereas 54 miRNAs, including 5 known miRNAs and 49 new miRNAs, were identified in the uterine library. Screening with four types of gene prediction software revealed 73 target genes associated with uterine involution, and subsequently, GO annotation and KEGG pathway analysis were performed. The results showed that, in the hypothalamic–uterine axis, uterine involution in postpartum ewes might primarily involve two miRNA-target gene pairs, namely, miRNA-200a–PTEN and miRNA-133–FGFR1, which can participate in GnRH signal transduction in the upstream hypothalamus and in the remodeling process at the downstream uterus, through the PI3K–AKT signaling pathway to influence the recovery of the morphology and functions of the uterus during the postpartum period in sheep. Therefore, identification of differentially expressed miRNAs in this study fills a gap in the research related to miRNAs in uterine involution in postpartum ewes and provides an important reference point for a comprehensive understanding of the molecular mechanisms underlying the regulation of postpartum uterine involution in female livestock.

## 1 Introduction

Recovery of the uterus to its pre-pregnancy state after childbirth is called uterine involution. Generally, postpartum uterine involution mainly includes morphological and physiological aspects; it is a process in which a series of changes in the morphology, structure, and function of the uterus of the sheep occur to restore it to the prenatal uterine state, which plays a crucial role in ensuring normal reproductive capacity and estrous cycle of the sheep after giving birth (Naznin et al., 2019; Ioannidi et al., 2020).

The hypothalamus, located at the base of the diencephalon and below the thalamus, links the nervous and endocrine systems through the pituitary gland (Neumann et al., 2019). Its function is to secrete gonadotropin-releasing hormones (e.g., GnRH and oxytocin) and gonadotropin-inhibitory hormones (e.g., GnIH) to stimulate or suppress gonadotropin levels produced in the anterior lobe of the pituitary (Silveira et al., 2017; Tsutsui and Ubuka, 2018; Kania et al., 2020). The hypothalamus–pituitary–ovary–uterus (HPOU) axis is a relatively independent system with the hypothalamus as its upstream starting point and the uterus as its downstream end, each playing an important role in the HPOU process. Normally, oxytocin (OXT) synthesized by the hypothalamus is released through the pituitary gland and circulates to the uterus to participate in regulation of delivery or in the downstream uterine involution process. At the same time, GnRH synthesized by the hypothalamus induces the pituitary gland to release follicle-stimulating hormone (FSH) and luteinizing hormone (LH), enters blood circulation, and is transported to the ovary to cause it to synthesize and release E${}_{2}$ and P${}_{4}$. It then acts on the uterine end to influence fertilization, embryo implantation, and cyclic morphological and functional changes at the uterine end. In contrast to the processes occurring in the hypothalamus, at the downstream uterine end, stimulation of the cervix by sexual intercourse induces secretion of prolactin (PRL) at peak levels in the supraoptic nucleus of the upstream hypothalamus and formation of a peak that lasts for two days in order to promote the secretion of ovarian progesterone during early pregnancy or pseudopregnancy, thus forming an intact pathway to target hypothalamus–pituitary–ovary (HPO) axis at the uterine end (Napso et al., 2018; Davis and LaVoie, 2019). In addition, OXT synthesized by the supraoptic and paraventricular nuclei of the hypothalamus can target the uterine end to stimulate the synthesis and secretion of prostaglandin (PG) hormone, which in turn induces the cyclic formation and degeneration of the corpus luteum tissue in the ovaries through the ovarian–uterine arteriovenous anastomosis branch to regulate the initiation and cyclic changes of the female estrous cycle. Therefore, in addition to the positive and negative feedback regulation of the HPO axis, there is also a direct or indirect network regulatory relationship between the hypothalamus and uterus; however, this regulatory relationship has rarely been reported, especially at the molecular level of miRNAs.

In recent years, with the development of high-throughput technology and bioinformatics technology, the study of miRNAs has become increasingly thorough and has further extended to the exploration of their regulatory mechanisms. At present, a large number of studies have found that there are abundant and stable miRNAs in smooth muscle cells, and these miRNAs play an important role in the proliferation, hypertrophy, and differentiation of the cells (Carletti and Christenson, 2009; Li, 2014; Nothnick, 2016). This suggests that miRNAs are also likely to play an important role in postpartum uterine involution, especially in the process of uterine smooth muscle contraction, thereby affecting the overall recovery of the uterus. Methylergonovine, a non-steroidal smooth muscle contraction agent, has widely been used in female livestock to stimulate the myometrium to increase its contraction force, frequency, and amplitude, which can effectively accelerate the process of uterine involution (Nworgu et al., 2010; Fanning et al., 2017). Based on this information, in this study, miRNA libraries were constructed for the hypothalamic and uterine tissues of sheep intramuscularly injected with methylergonovine and sheep intramuscularly injected with saline postpartum using miRNA HiSeq deep sequencing technology to determine differentially expressed miRNAs related to the uterine involution process in postpartum ewes and to lay a solid foundation for in-depth analysis of the molecular network regulatory mechanisms of mammalian uterine involution.

## 2 Materials and methods

### 2.1 Animals and experiment design

A total of 40 adult Kazakh ewes of similar age (3–4 weeks) and weight (45–50 kg), without uterine diseases and with normal reproductive performance, were selected and fed uniformly at the Shihezi University Experiment Station within the same breeding environment. The model of postpartum uterine involution was constructed using methylergonovine (treatment group) and normal saline (control group) intramuscular injection to ensure that the uterine involution of sheep postpartum had relatively obvious phenotypic changes. In the treatment group, 15 ewes were injected with 0.2 mg ergometrine in the inner thigh of the hind limb on the first day postpartum; in the control group, 15 ewes were injected with the same volume of normal saline in the inner thigh of the hind limb on the first day postpartum. Subsequently, the dynamic changes in the uterus were monitored daily. In addition, an early weaning program was carried out on the seventh day postpartum by separating the ewes from their lambs (the lambs were moved to another enclosure for milk replacer feeding).

### 2.2 Sample collection

The process of uterine involution was monitored using a Tringa Vet veterinary portable ultrasound system, and uterine involution was judged to be complete when the uterine cavity was completely closed, no residual fluid was found in the uterine cavity, and the diameter of the uterus was less than 2 cm. Then, three ewes meeting the above conditions were selected and recorded as the group with faster uterine involution treated with methylergonovine (UF) and the group with slower uterine involution treated with normal saline (US). After slaughtering, hypothalamic and uterine tissues were collected, numbered, and stored in liquid nitrogen for further analysis.

### 2.3 Total RNA extraction and quality testing

The total RNA of each sample was extracted using chloroform, isopropanol, and 70 % ethanol; the total RNA of the UF and US samples were then placed on dry ice and sent to Beijing Honortech Technology Co., Ltd. The RNA integrity and purity were tested using the Agilent 2100 Bioanalyzer, Kaiao K5500 micro-spectrophotometer, and the Agilent RNA 6000 Nano Kit, and qualified samples were used to construct hypothalamic and uterine miRNA libraries.

### 2.4 MiRNA library construction and testing

The above-constructed miRNA libraries of the postpartum sheep hypothalamus and uterus in the UF and US groups were recorded as faster uterine involution–hypothalamus (UFH) vs. slower uterine involution–hypothalamus (USH) and faster uterine involution–uterus (UFU) vs. slower uterine involution–uterus (USU). Subsequently, the Agilent 2100 Bioanalyzer and ABI StepOnePlus Real-Time PCR System were used to check the quality and yield of the constructed libraries. Finally, the sequencing libraries were subjected to high-throughput sequencing using the Illumina HiSeq 2500 platform according to the SE50 sequencing strategy.

### 2.5 Bioinformatics analysis

The different sequences obtained were first screened to obtain plausible target sequences, and the quality, length, and common sequences between samples were noted for these sequences. Then, the target sequences were annotated by classification to obtain information on the components and expression levels in the samples, and new miRNA predictions were made for the unannotated small RNA fragments. Meanwhile, the miRNAs that were differentially expressed between the different groups in the above libraries were analyzed and screened using a log2 ratio and a scatterplot, respectively. Finally, the screened differentially expressed known miRNAs and new miRNAs were subjected to cluster analysis, target gene prediction and GO (http://geneontology.org/, last access: 10 December 2020) functional annotation of target genes, and KEGG (http://www.genome.jp/kegg/, last access: 10 December 2020) pathway annotation. Genes with Hochberg false discovery rate (FDR) ≤0.05 were considered as significantly enriched as target gene candidates.

### 2.6 Validation of quantitative real-time polymerase chain reaction (qRT-PCR)

Twelve miRNAs, with six miRNAs in the hypothalamus and uterus libraries each, were randomly selected to verify the reliability of the sequencing results. miRNAs and mRNAs were reverse-transcribed to cDNA with the miRcute miRNA First-strand cDNA Kit (TIANGEN, Beijing, China). qRT-PCR was then performed using the MX3000p qRT-PCR System (Stratagene, USA) and miRcute miRNA Premix SYBR (TIANGEN), following the manufacturer's instructions. All reactions were performed in triplicate. U6 RNA was chosen as an endogenous internal control, and the relative expression levels were calculated based on the 2${}^{-\Delta \Delta \mathrm{Ct}}$ method. The miRNA-specific primers are listed in Table S1A and B in the Supplement.

### 2.7 Differential expression of miRNA-target gene pairs in hypothalamus–uterus axis

Based on the joint analysis of all miRNA libraries, the proposed candidate miRNAs and potential target genes were finally selected, and total RNA was extracted from different tissues of the same as the UF vs. US group and batch for real-time fluorescence detection of miRNAs and mRNA. These potential target genes primers are listed in Table S1C. All experiments were repeated three times with three replicates for each sample.

## 3 Results and analysis

### 3.1 Detection of morphological changes in uterine involution

**Figure 1:**
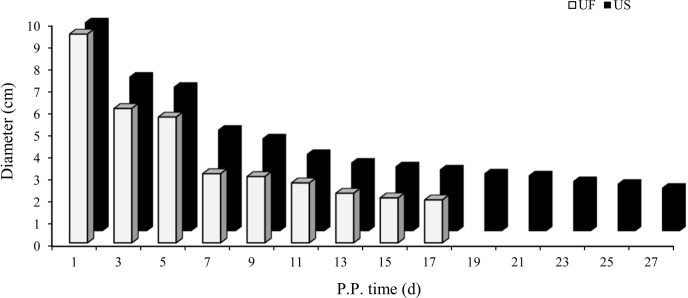
Trends for maximum cross-sectional diameter of the postpartum uterine horn between UF and US group.

B-mode ultrasonography is a relatively effective and reliable means of monitoring the state of uterine involution in sheep after delivery. Two days after delivery, the uterine horns were large and contained a high amount of lochia. Linear array probe B-mode ultrasonography was used for transrectal examination of the changes in uterine involution after delivery, and the overall state of the uterus could not be completely observed. Starting from the third day postpartum in the treatment group, the results showed that the uterine horns of the ewes showed obvious retraction; however, after the cervix and uterine body were completely closed, the uterine cavity in the uterine horns was still in a partially opened state, indicating that the completion of uterine horn recovery could be used as a marker of overall completion of uterine involution. Based on the above uterine involution completion evaluation criteria, specific B-mode ultrasonography tracking was used to monitor the trend of changes in maximum cross-sectional diameter during the recovery of the uterine horns in postpartum ewes, as shown in Fig. 1. The results showed that the completion time of uterine horn recovery was 17 d in the UF group and 27 d in the US group. The phenotypic completion time of uterine involution in these two groups was obviously different, which confirmed that the animal model of uterine horn recovery was successfully constructed for different time periods and could be used for further experiments.

### 3.2 Identification and analysis of sequence quality pre-processing

To ensure the accuracy of the test data and improve the quality of data acquisition, the final clean reads of the two samples were obtained based on the annotation information and data pre-processing. A total of 42 412 012 and 41 768 134 raw reads were obtained from the UFH and USH libraries, respectively. Similarly, a total of 42 355 026 sequences (raw reads) were obtained from the UFU library, and 40 960 450 sequences were obtained from the USU library. After adapter and contaminated sequence trimming, as well as pretreatment of clean data and the annotated RFam and miRbase databases, 41 120 980 and 40 666 618 high-quality sequences (clean reads) were obtained from UFH and USH, respectively, accounting for 96.96 % and 97.36 %, respectively, of the total number of sequences (Table 1); similarly, 40 978 134 and 39 652 778 high-quality sequences were obtained from the UFU and USU libraries, respectively, accounting for 96.75 % and 96.81 %, respectively, of the total number of sequences (Table 1). All these sequences indicated that the miRNA information libraries constructed in this experiment were of good quality and could be used for further analysis.

**Table 1:** Percentage and distribution of sequencing results in UF vs. US libraries.

Reads type	Raw reads	Clean reads	
		Number	(Clean reads/
			Raw reads) %
UFH	42 412 012	41 120 980	96.96 %
USH	41 768 134	40 666 618	97.36 %
UFU	42 355 026	40 978 134	96.75 %
USU	40 960 450	39 652 778	96.81 %

### 3.3 Taxonomic annotation of small RNA sequences

**Figure 2:**
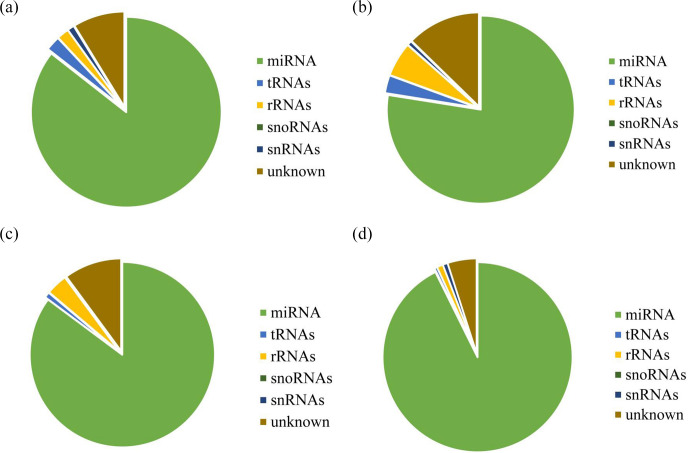
Statistics of the distribution characteristics of non-coding small RNAs between UF and US group.

After decontamination, all obtained clean-read sequences measuring over 18 nt were used for genomic localization and taxonomic annotation. The small RNA sequences were then analyzed by comprehensive alignment with non-coding small RNAs, RNA repeats, introns, and exons from Rfam database 11.0 and with NCBI GenBank database, and the results are shown in Fig. 2. The results of the alignment revealed that UFH (Fig. 2a), USH (Fig. 2b), UFU (Fig. 2c), and USU (Fig. 2d) information bases were mostly miRNAs, and known and unknown miRNAs from both information bases were used as data sources for subsequent analyses.

### 3.4 Differential miRNAs expression analysis

**Figure 3:**
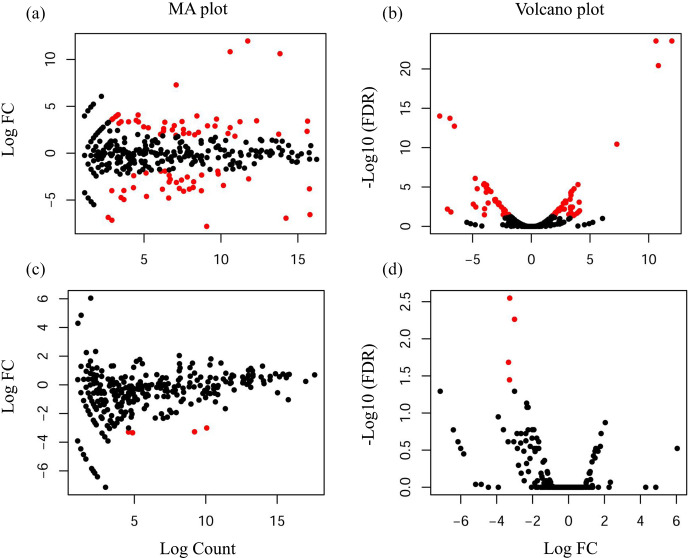
Differentially expressed known miRNAs were identified by deep sequencing. A MA plot **(a, c)** and a volcano plot **(b, d)** were used to display the differentially expressed miRNA patterns in UF vs. US libraries. One point as one miRNA, red points in the plot represent differentially expressed miRNAs, and black points show no statistically significant differences in the expression of miRNAs between UF and US libraries.

Compared with the US group, 118 significantly differentially expressed miRNAs, including 33 known miRNAs and 85 unknown miRNAs, were found in the UFH library. Among the 33 known miRNAs, 15 were up-regulated and 18 were down-regulated. Some of the known miRNAs with differential expression are listed in Fig. 3a and b. Furthermore, 54 significantly differentially expressed miRNAs, including 5 known miRNAs and 49 unknown miRNAs, were found in the UFU library. Among the 5 known miRNAs, 2 were up-regulated and 3 were down-regulated. Some of the known miRNAs with differential expression are listed in Fig. 3c and d. Notably, we found four common differentially expressed miRNAs in the hypothalamic and uterine libraries, namely, oar-miR-665-3p, oar-miR-379-5p, oar-miR-200a, and oar-miR-133. In addition, the positions and maturation sequences in the genome of the six unknown miRNAs showing the most significant differential expression in the four libraries mentioned above are listed in Table 2.

**Table 2:** Relatively higher abundance of six novel miRNA positions on the genome and its sequence in UF vs. US libraries.

Groups	miRNA	Mature sequence (5${}^{\prime }$–3${}^{\prime }$)	Chromosome	Free energy
			localization	kcal/mol
UFH vs. USH	oar-novel-miR-295-5p	UGAUUGUCCAAACGCAAUUCUC	Chr 20: 7496785-7496849	-26.3
	oar-novel-miR-1414-5p	UGAUUGUCCAAACGCAAUUCU	Chr 20: 7496785-7496846	-26.3
	oar-novel-miR-1168-3p	UCUUUGGUUAUCUAGCUGUAU	Chr 1: 104964304-104964378	-25.9
	oar-novel-miR-440-3p	UCUUUGGUUAUCUAGCUGUAUGA	Chr 1: 104964302-104964380	-25.7
	oar-novel-miR-1119-5p	GUGGACUUCCCUGGUAGCUCAGC	Chr 2: 157399570-157399661	-40.2
	oar-novel-miR-1151-5p	UCUGGCUCCGUGUCUUCACUCCCG	Chr 1: 1050720-1050782	-30.5
UFU vs. USU	oar-novel-miR-555-5p	UGGACGGAGAACUGAUAAGGGU	Chr 18: 24440152-24440240	-26.1
	oar-novel-miR-1185-3p	UGGAAUGUAAAGAAGUAUGUAU	Chr 23: 34763997-34764058	-20.9
	oar-novel-miR-719-5p	CCGCGGCGGGGGCGGUCC	Chr 3: 221307563-221307632	-38.4
	oar-novel-miR-378-3p	UAACUGUGGCGCAUGGGCUUCA	Chr 2: 11892272-11892346	-27.5
	oar-novel-miR-1119-5p	GUGGACUUCCCUGGUAGCUCAGC	Chr 2: 157399570-157399661	-40.2
	oar-novel-miR-1210-3p	UUAUUGCUUAAGAAUACGCGUAGU	Chr 1: 74528676-74528737	-24.6

### 3.5 Validation of library establishment by qRT-PCR

**Figure 4:**
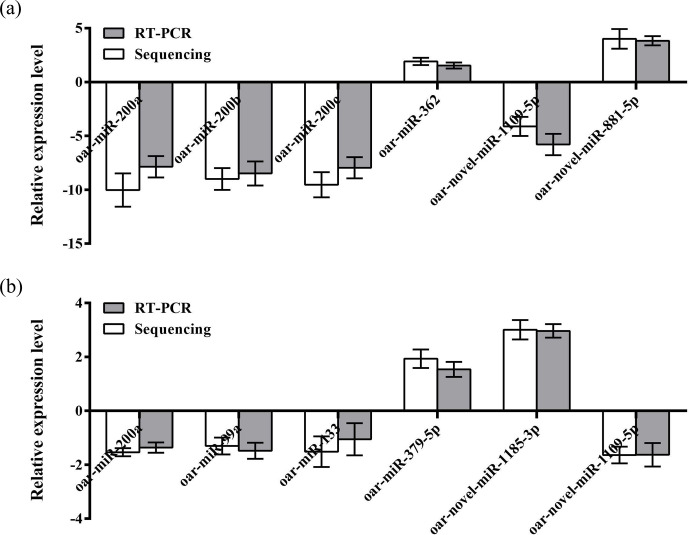
Validation of the Solexa sequencing results in the hypothalamic and uterine libraries. Data are mean ± SEM.

To further verify the reliability of the four miRNA libraries that had been constructed, six differentially expressed miRNAs, including four known miRNAs and two unknown miRNAs, were randomly selected from the hypothalamic and uterine libraries, respectively, for qRT-PCR validation in this experiment. U6 was used as the internal reference gene. The relative expression levels of the above miRNAs were calculated using the 2${}^{-\Delta \Delta \mathrm{Ct}}$ method, as shown in Fig. 4a and b. The results showed that the trend of the differential expression levels of the six randomly selected miRNAs in the hypothalamic and uterine libraries was consistent with the high-throughput variability of library sequencing, which confirmed that the data in the hypothalamic and uterine libraries were authentic.

### 3.6 Screening and analysis of candidate target genes

**Figure 5:**
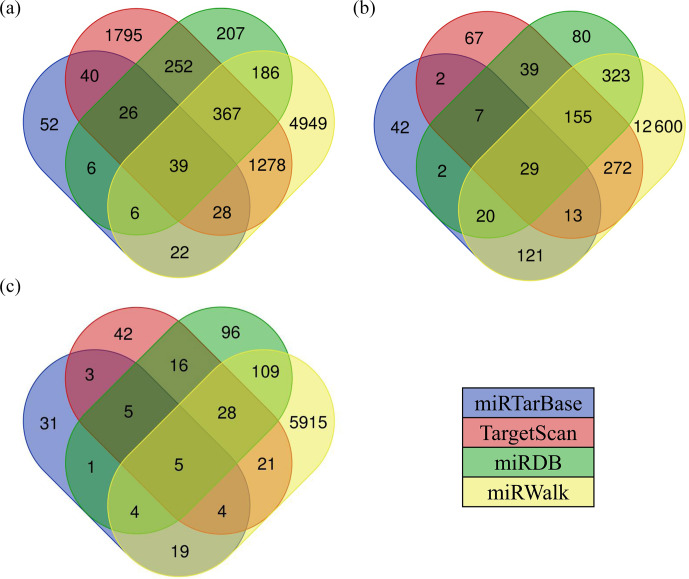
Various types of software for predicting the target genes of miRNAs. The numbers in the intersection of the four circles refer to the candidate target genes of miRNAs.

There are various types of software for predicting the target genes of miRNAs. In this study, we selected four types of software, including TargetScan, miRDB, miRWalk, and miRTarBase to predict the target genes of miRNAs, and finally chose the intersection of target genes as the candidate target genes of miRNAs. The results showed that the number of target genes for differentially expressed miRNAs common between the hypothalamic and uterine libraries was 39 for oar-miR-200a (Fig. 5a), 29 for oar-miR-133 (Fig. 5b), and 5 for oar-miR-379-5p (Fig. 5c); however, oar-miR-665-3p was not found to have an intersectional target gene. The above miRNA-target gene pairs were used for subsequent GO annotation entries and KEGG Pathway enrichment analysis.

### 3.7 Candidate target genes GO and KEGG analysis

**Figure 6:**
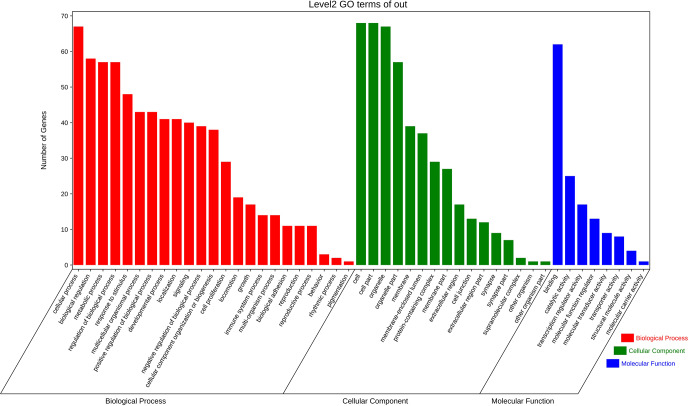
Seventy-three target genes were analyzed using taxonomic annotation and enrichment analysis in terms of molecular function, biological process, and cell composition.

**Figure 7:**
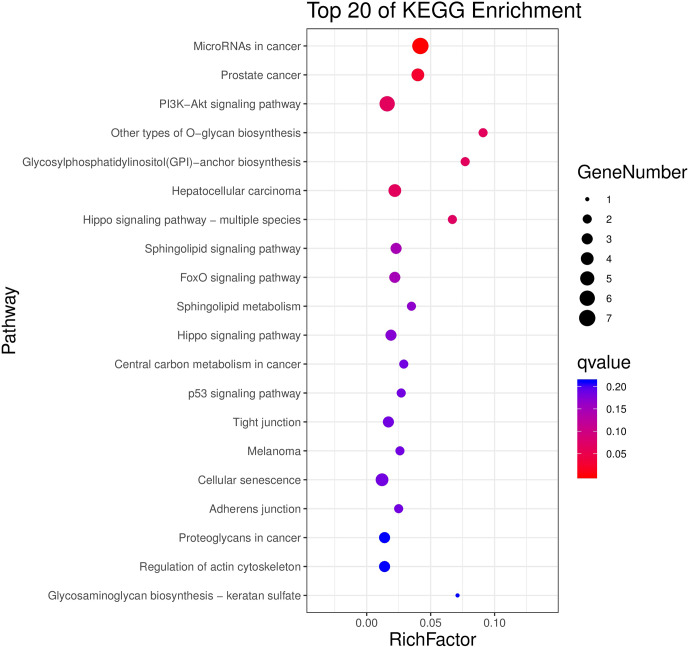
Seventy-three target genes were conducted using KEGG signaling pathway enrichment analysis. P value <0.05 was considered statistically significant.

The above-mentioned 73 target genes were analyzed using taxonomic annotation and enrichment analysis in terms of molecular function, biological process, and cell composition (as shown in Fig. 6). The results showed that the target genes in this study were involved in a large number of biological regulatory processes, such as reproduction, reproductive process, rhythmic process, multi-organism process, growth, cell proliferation, and positive/negative regulation biological process. At the same time, KEGG signaling pathway enrichment analysis of the aforementioned genes was performed (Fig. 7); the results revealed that a large number of candidate target genes were significantly enriched in several pathways, such as the PI3K–AKT signaling pathway. Here, we particularly note that the PI3K–AKT signaling pathway, Hippo signaling pathway, and glycosylphosphatidylinositol (GPI)-anchor biosynthesis signaling pathway are involved in and play a bridging role in many biological regulatory processes.

### 3.8 Variance analysis of miRNA-target gene expression in hypothalamus–uterus axis

**Figure 8:**
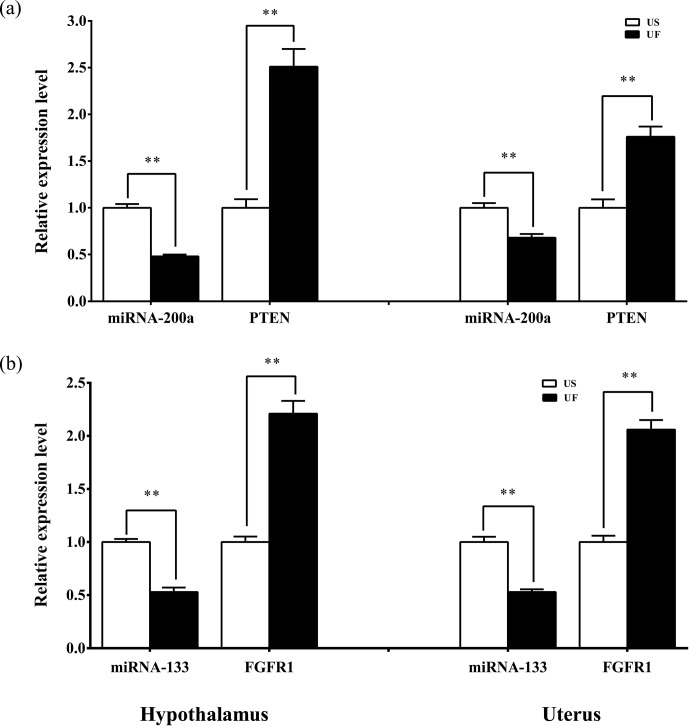
Expression levels of the candidate miRNAs and their target genes in the hypothalamic and uterine samples. All the samples are consistent with the library samples.

Based on the aforementioned miRNA target prediction as well as GO and KEGG enrichment analyses of target genes, we noted the presence of two miRNA-targeted gene pairs in PI3K–AKT, namely, miRNA-200a–PTEN and miRNA-133–FGFR1, which are likely to play important roles in the upstream hypothalamic GnRH signaling and downstream uterine remodeling processes. To further verify the reliability of the above screening results, tissue samples from the same treatment group were subsequently processed using qRT-PCR (Fig. 8). The results showed that miRNA-200a and its target gene PTEN (Fig. 8a) as well as miRNA-133 and its target gene FGFR1 (Fig. 8b) were significantly differentially expressed in both hypothalamic and uterine tissues, and the expression trend between the two showed a clear negative correlation. This suggests that there is a typical negative regulatory relationship between the miRNAs and target genes, which guides the direction of and lays the foundation for further research on the functional regulatory mechanisms of mammalian uterine involution.

## 4 Discussion

The gestation period of ewes is 5 months, making it theoretically feasible for ewes to have two litters per year. However, to increase the frequency of lambing in actual production, the ovine uterus must be restored to its prenatal state as soon as possible, and a favorable intrauterine environment must be created for the fertilized egg to implant, the embryo to develop, and the fetus to mature. Therefore, the length of the postpartum maternal uterus recovery directly restricts the breeding cycle and production performance of the ewes, which plays an important role as a “checkpoint” to ensure the production efficiency and economic benefits of large-scale sheep farms.

The hypothalamus, which is the hub that regulates the endocrine activities in mammalian organisms, integrates signals from the brain and periphery and responds in a timely and appropriate manner. Particularly, being at the origin of the HPOU axis, it plays a central regulatory role in the mechanism of maternal uterus recovery. The uterus, at the terminal end of the HPOU axis, may not be as complex as the hypothalamus in terms of intrinsic molecular signal regulation; however, the changes in its morphological structure and function, compared with those in the hypothalamus, can more directly reflect the changes in maternal endocrine function after delivery, especially in uterine involution. Numerous studies have found that miRNAs are widely expressed in both the hypothalamus and uterus, and they can regulate the spatial and temporal expression of the corresponding target genes in both the hypothalamus and uterus to make their respective tissues respond to the regulatory signals upstream of or feedback signals downstream of them. For example, using a cellular model, Lannes et al. (2016) found that in the hypothalamus, miR-132/212 regulated the synthesis and secretion of GnRH through the SIRT1–FOXO1 signaling pathway and stimulated FSH secretion to influence the function and activity of the hypothalamic–pituitary–gonadal (HPG) axis (Lannes et al., 2015). Additionally, they showed that miR-125b could also regulate the efficacy of GnRH signaling in the hypothalamus by targeting G<q/11 signals. However, miR-125b was inhibited by miR-132 to form a regulatory network circuit involved in the action and regulation of GnRH (Lannes et al., 2016). Similarly, Choi et al. (2013) showed that miR-24 can target and regulate the synthesis of OXT in the paraventricular and supraoptic nuclei of the hypothalamus and is involved in the hierarchical regulation of the entire HPOU axis by mediating the synthesis and secretion of pituitary hormones. Nothnick (2008) reported that in the uterus, miRNA-204 participates in the regulation of labor and postpartum uterine tissue changes by modulating uterine MMP9 gene expression and influencing the endometrium thickness; additionally, Williams et al. (2012) showed that miR-199a inhibits COX-2 gene expression in uterine tissue and influences cyclicity changes among PGF${}_{2\alpha }$, P${}_{4}$, and E${}_{2}$ in the maternal body to maintain the resting state and contraction of the uterus (Williams et al., 2012). However, in the present study, we found four intersecting miRNAs in the aforementioned two libraries, namely miR-200a, miR-665-3p, miR-379-5p, and miR-133, through comparative analysis of differential miRNAs in hypothalamic and uterine libraries. We also predicted 73 target genes of these miRNAs using four types of software, including TargetScan, miRDB, miRWalk, and miRTarBase. Using GO annotation and KEGG signaling pathway enrichment analysis, we found that two miRNA-target gene pairs, namely miRNA-200a–PTEN and miRNA-133–FGFR1, are involved in PI3K–AKT signaling, which are likely to be involved in the regulation of postpartum uterine involution in hypothalamic and uterine tissues. PTEN is a multifunctional phosphatase that regulates its own activity through its C-terminal phosphorylation and is involved in pro-apoptotic or apoptotic processes, thereby regulating downstream PI3K/AKT signaling. PTEN is preferentially expressed in most neurons and regulates neuronal differentiation and synapse formation, playing an important role in the central nervous system (CNS). Thus, PTEN may also be involved in regulating the activity of GnRH neurons in the hypothalamic nervous system, which in turn affects the transmission and regulation of signals downstream of the HPOU axis. Noteworthily, while studying uterine trophoblast invasion, maternal vascular remodeling, and epithelial and myometrial hyperplasia, Laguë et al. (2010) found that at the uterine end downstream of the HPOU axis, PTEN also plays an important regulatory role in uterine tissue (Laguë et al., 2010). This suggests that PTEN may also have a key regulatory role in the postpartum uterine remodeling process, including uterine involution and restoration of the intrauterine environment. However, FGFR-1 belongs to the FGF family and is important in the regulation of numerous CNS functions, including neural induction, cell fate, neuronal survival, axonal growth, and cell migration. When studying the FGFR1 subtypes, Chung et al. (2008) found that the number of GnRH neurons in the homozygous FGFR1 subtype was 88 % less than that in the wild type, suggesting that FGFR1 is very likely to affect secretion by GnRH neurons by altering the number of GnRH neurons in the hypothalamus, thereby regulating the HPOU axis signaling (Chung et al., 2008). Similarly, in the downstream signaling in the uterus, Sangha et al. (1997) showed that FGFR1 plays a key role in regulating the “maturation” of the endometrium and regeneration of normal endometrium through localization of endometrial FGFR1 (Sangha et al., 1997). This indicates that FGFR1 in uterine tissues is likely to be directly involved in the regulation of the recovery process of the uterus after delivery, including the regeneration, repair, and maturation of the endometrium. Thus, several differentially expressed miRNAs are present in both the upstream hypothalamic end and the downstream uterine end in the process of postpartum uterine involution, which could be involved in regulation of postpartum uterine involution through their corresponding target genes acting directly on the uterus or indirectly on the hypothalamus.

## 5 Conclusions

In conclusion, we successfully screened out four common differentially expressed miRNAs in ewes by constructing their hypothalamic and uterine miRNA libraries. In addition, through comprehensive bioinformatics analysis, we identified two pairs of miRNA-target genes, namely miRNA-200a–PTEN and miRNA-133–FGFR1, which may directly act on uterine tissue or indirectly on the hypothalamus through the PI3K–AKT signaling pathway during postpartum uterine involution to regulate postpartum uterine morphological and functional recovery. This provides an important reference for further studying the regulatory process of uterine involution in postpartum livestock and in-depth analysis of its molecular network regulatory mechanisms.

## Supplement

10.5194/aab-64-119-2021-supplement The supplement related to this article is available online at: https://doi.org/10.5194/aab-64-119-2021-supplement.

## Data Availability

The data are available from the corresponding author upon request.
