# Supplementary material for: Identification and validation of key miRNAs and miRNA–mRNA regulatory network associated with uterine involution in postpartum Kazakh sheep
Source: Arch Anim Breed. 2021 Apr 23;64(1):119–29. doi: 10.5194/aab-64-119-2021 (PMC8131964; doi:10.5194/aab-64-119-2021)
Supplement: The supplement related to this article is available online at: https://doi.org/10.5194/aab-64-119-2021-supplement. [file aab-64-119-supplement.pdf]

Supplement of Arch. Anim. Breed., 64, 119–129, 2021
https://doi.org/10.5194/aab-64-119-2021-supplement
© Author(s) 2021. CC BY 4.0 License.

Open Access
Archives Animal Breeding

Supplement of

Identification and validation of key miRNAs and miRNA–mRNA regulatory network associated with uterine involution in postpartum Kazakh sheep

Heng Yang et al.

Correspondence to: Heng Yang (yh20183007@swu.edu.cn) and Huihao Xu (xuhuihao2dai@163.com)

The copyright of individual parts of the supplement might differ from the article licence.

Table S1A the primer sequence of miRNA (hypothalamus)

	miR_name	Primer
UFH/USH	oar-miR-200a	GGGGTAACACTGTCTGGTAACGATGTT
	oar-miR-200b	GGGGTAATACTGCCTGGTAATGATG
	oar-miR-200c	GGTAATACTGCCGGGTAATGATGGA
	oar-miR-665-3p	CGGGCCATAATACATGGTTAACC
	oar-miR-362	GCAATCCTTGGAACCTAGGTGT
	oar-novel-miR-1109-5p	GGCCCGTCCCGTGCGTCAA
	oar-novel-miR-881-5p	CTGGCTCCGTGTCTTCACTCC
	U6	CAAGGATGACACGCAAATTCG

Table S1B the primer sequence of miRNA (uterus)

	miR_name	Primer
UFU/USU	oar-miR-200a	GGGGTAACACTGTCTGGTAACGATGTT
	oar-miR-99a	CCGAACCCGTAGATCCGATCTTG
	oar-miR-133	TCCGTTTTGGTCCCCTTCAAC
	oar-miR-379-5p	CGTGGTAGACTATGGAACGTAGGC
	oar-novel-miR-1185-3p	CCCGGCCTGGAATGTAAAGAAGTATGTAT
	oar-novel-miR-1109-5p	GGCCCGTCCCGTGCGTCAA
	U6	CAAGGATGACACGCAAATTCG

Table S1C the primer sequence of target genes

	Gene	Primer (5'–3')	Product size (bp)
UF/US	PTEN	F: CACACGACGGGAAGACAAGT<br>R: AGGTTTCCTCTGGTCCTGGTA	167
	FGFR1	F: CAAACCAAACCGTATGCCCG<br>R: ACCTTGTAGCCTCCGATCCT	192
	β-catenin	F: GGGAGTCCGCATGGAAGAAA<br>R: GCAGCTGCACAAACAATGGA	122
